# The Use of Aspirin in the Treatment of Glycosuria and Diabetes Mellitus

**Published:** 1903-01-17

**Authors:** 


### THE USE OF ASPIRIN IN THE TREATMENT OF GLYCOSURIA AND DIABETES MELLITUS.

Dr. T. R. Williamson,[1] of Manchester, points out that in certain cases of diabetes and glycosuria the patients cannot take sodium salicylate in large doses on account of the gastric disturbance, toxic effects, etc., produced. In such cases he has usually found that aspirin can be taken without causing these bad effects, for it can in many cases be taken in large doses for long periods much better than sodium salicylate. In some cases again he has given both drugs—sodium salicylate several times a day and aspirin as a powder at night — with advantage. Aspirin is a salicylate compound (acetyl-salicylic acid), which is stated to be decomposed only when it reaches the small intestine; and it is said to produce none of the toxic symptoms which are sometimes caused by sodium salicylate. In the four cases which Dr. Williamson records he says that the diminution of the sugar excretion could have been caused only by the action of the aspirin, and the good results were not associated with the occurrence of complications, gastric disturbance, feeling of depression, or other unfavourable symptoms. As is the case with salicylates, the drug has most influence in the less severe forms of the disease. The dose required to produce a decided diminution of the sugar in the less severe forms of diabetes is rather large—15 grains, four, five, or sometimes six times a day; and the drug requires to be watched, for in spite of statements to the contrary, it does sometimes produce noises in the ears and other toxic symptoms. These, however, occur less frequently than with the salicylates. Aspirin may be given as a powder, to be taken in a tablespoonful of water, to which one or two drops of lemon juice have been added. Unless administered in a slightly acid fluid it is apt to give rise to much gastric disturbance—one of the very things for the sake of avoiding which its use is recommended.

[1] Brit. Med. Jour., Dec. 27.

